# *Ex Vivo* Maintenance of Primary Human Multiple Myeloma Cells through the Optimization of the Osteoblastic Niche

**DOI:** 10.1371/journal.pone.0125995

**Published:** 2015-05-14

**Authors:** Wenting Zhang, Yexin Gu, Qiaoling Sun, David S. Siegel, Peter Tolias, Zheng Yang, Woo Y. Lee, Jenny Zilberberg

**Affiliations:** 1 Department of Chemical Engineering and Materials Science, Stevens Institute of Technology, 1 Castle Point on Hudson, Hoboken, New Jersey, 07030, United States of America; 2 John Theurer Cancer Center, Hackensack University Medical Center, Hackensack, New Jersey, 07601, United States of America; 3 Department of Chemistry, Chemical Biology, and Biomedical Engineering, Stevens Institute of Technology, Hoboken, New Jersey, 07030, United States of America; 4 Center for Healthcare Innovation, Stevens Institute of Technology, Hoboken, New Jersey, 07030, United States of America; 5 Research Department, Hackensack University Medical Center, Hackensack, New Jersey, 07601, United States of America; Chang Gung University, TAIWAN

## Abstract

We previously reported a new approach for culturing difficult-to-preserve primary patient-derived multiple myeloma cells (MMC) using an osteoblast (OSB)-derived 3D tissue scaffold constructed in a perfused microfluidic environment and a culture medium supplemented with patient plasma. In the current study, we used this biomimetic model to show, for the first time, that the long-term survival of OSB is the most critical factor in maintaining the *ex vivo* viability and proliferative capacity of MMC. We found that the adhesion and retention of MMC to the tissue scaffold was meditated by osteoblastic N-cadherin, as one of potential mechanisms that regulate MMC-OSB interactions. However, in the presence of MMC and patient plasma, the viability and osteogenic activity of OSB became gradually compromised, and consequently MMC could not remain viable over 3 weeks. We demonstrated that the long-term survival of both OSB and MMC could be enhanced by: (1) optimizing perfusion flow rate and patient-derived plasma composition in the culture medium and (2) replenishing OSB during culture as a practical means of prolonging MMC’s viability beyond several weeks. These findings were obtained using a high-throughput well plate-based perfusion device from the perspective of optimizing the *ex vivo* preservation of patient-derived MM biospecimens for downstream use in biological studies and chemosensitivity analyses.

## Introduction

Multiple myeloma (MM), an incurable B-cell malignancy, is the second most common blood cancer in the U.S. with a typical survival of 5 to 7 years.[1,2] MM represents a paradigm for the intricate role played by the tumor microenvironment in the progression and development of drug resistant cancers. One of the major challenges associated with studying MM and evaluating new therapeutic approaches has been the lack of clinically relevant, high-throughput, and inexpensive in vivo and in vitro models. Primary human MM cells (MMC) rarely metastasize to murine and other animal bones due to species-related issues, resulting in the use of complicated, inconsistent, time-consuming, and costly patient derived models.[3,4] Of note, Lawson et al., [5] recently developed a new xenograft model where NOD/SCID-GAMMA (NSG) mice were injected via the tail vein with MM cell lines or with MMC from one patient sample. This simple approach proved beneficial for the assessment of various drug treatments. In terms of developing clinically relevant in vitro models for MM and other diseases, the use of primary cells has recently emerged as a critical issue since: (1) immortalizing human cells into cell lines by gene transfection perturbs the cells’ gene expression profiles and cellular physiology[6–8] and (2) cell lines do not capture cell heterogeneity.[9] However, ex vivo maintenance and expansion of primary human MMC have been problematic[10] due to the lack of an in vitro technology capable of reproducing the complex bone marrow microenvironment.

We recently reported the feasibility of engineering a patient-specific multiple myeloma tissue model for the ex vivo culture of primary MMC.[11] In this approach, bone marrow mononuclear cells (BMMC) were harvested from patients and cultured with: (1) an osteoblast (OSB)-derived 3D tissue scaffold constructed in a perfused microfluidic environment and (2) a culture medium supplemented with patient-derived plasma. Under this reconstructed MM environment, CD38^+^CD56^+^ MMC and CD138^+^ MMC populations were able to undergo up to 7 rounds of division in a 3-week culture.[11] In comparison to recent approaches by other investigators to recapitulate the MM microenvironment using biomimetic 3D scaffolds,[12–14] the significance of our approach is with its ability to provide perfusion.

Using this culture approach, we observed that: (1) the OSB-derived 3D tissue scaffold was primarily responsible for the ex vivo survival of MMC; (2) OSB’s long-term viability became gradually compromised during coculure with clinical bone marrow samples; and (3) medium flow rate and patient plasma concentration supported the ex vivo proliferation of MMC, presumably by influencing the above MMC-OSB interactions. Because of the well-recognized mechanosensing properties of OSB,[15–17] we hypothesize that perfusion is an important factor in regulating the growth of OSB and hence, the interplay between MMC and OSB. In regards to the plasma concentration effect, it has been postulated that aside from direct contact with OSB,[18] MMC growth is supported by soluble factors secreted by OSB such as interleukin 6 (IL-6) and Dickkopf-related protein 1 (DKK-1).[19,20] Furthermore, it is believed that MMC can also induce impaired OSB’s osteogenenic activity through the secretion of a number of soluble growth factors including DKK-1, interleukin-3 (IL-3), and interleukin-7 (IL-7).[21] In our culture system, patient plasma is likely supplying a number of these soluble constituents.

Based on these prior observations, the specific aims of this study were to: (1) establish the long-term survival of OSB and their direct contact with MMC, as the key important tumor niche factor enabling the ex vivo viability and proliferative capacity of primary patient-derived MMC; (2) systematically evaluate the effects of perfusion and plasma concentration on MMC-OSB interactions; and (3) test the replenishment of OSB during culture as a practical means of prolonging MMC’s viability and expansion beyond several weeks. As part of the first aim, we investigated the role of N-cadherin in mediating adhesion between primary MMC and OSB using genetically modified OSB with downregulated N-cadherin expression. It has been reported that N-cadherin, a mesenchymal cadherin associated with epithelial-to-mesenchymal transition, is: (1) aberrantly expressed by malignant plasma cells in MM patients[22,23] and (2) known to function as an adhesion molecule for homophilic binding between metastasized prostate cancer cells and OSB in the bone marrow of castration resistant prostate cancer tumors.[24] The resulting interaction between N-cadherin expressing OSB and prostate cancer cells are stipulated to promote osteoclastogenesis as well as drug resistance.[24,25] The second and third aims were conducted in the context of optimizing the ex vivo preservation of primary MMC from clinical biospecimens for downstream use in biological studies and chemosensitivity analyses.

## Materials and Methods

### Culture devices

**Fig 1:**
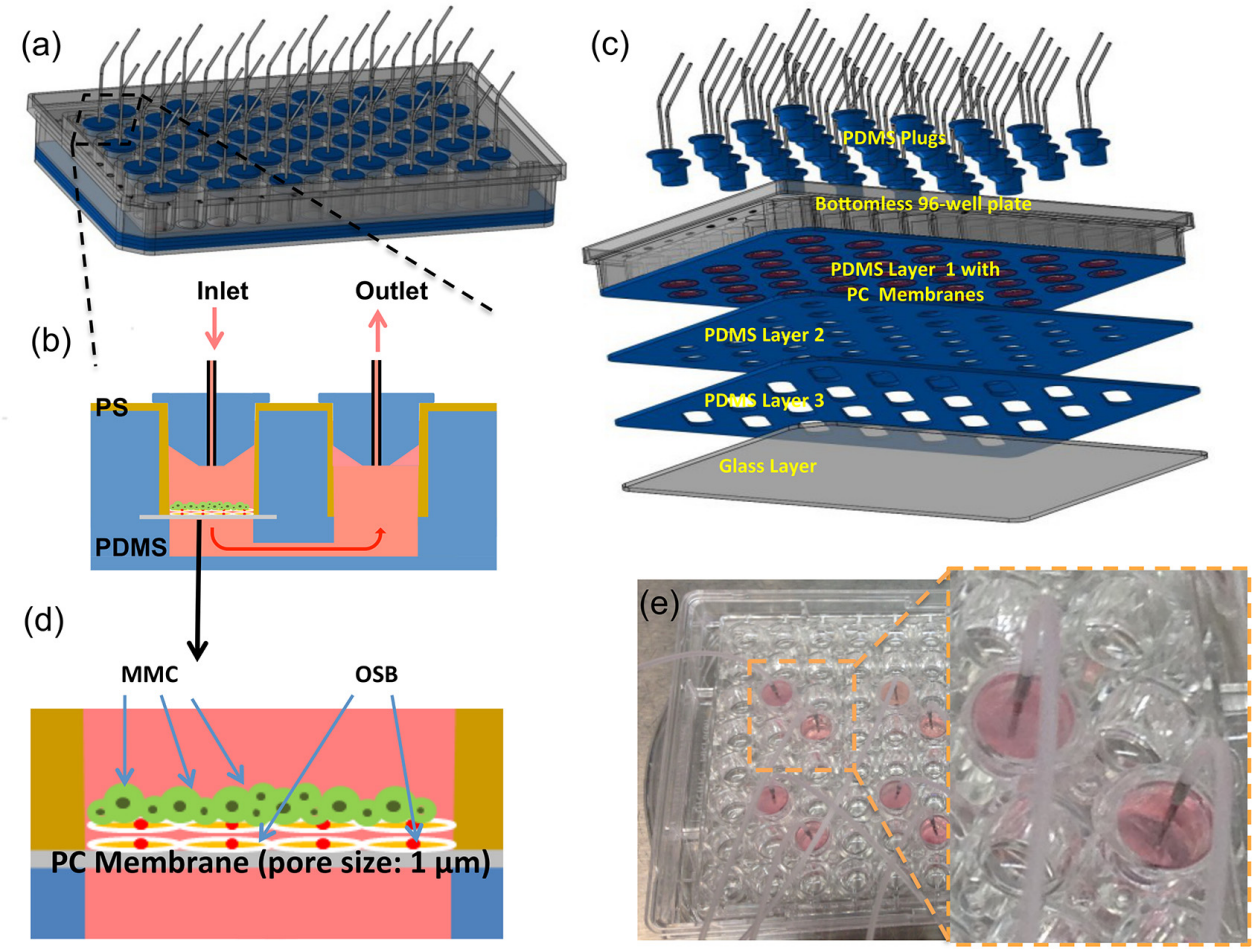
96-well plate-based perfusion device. (a)–(c) schematic representations of device design features and fabrication steps. (d) Schematic illustration of BMMC seeding onto OSB scaffold. (e) Actual device.

The 96-well plate-based perfusion device is shown in Fig 1E. As illustrated in Fig 1A, the device was assembled on a commercial available polystyrene (PS) bottomless 96-well plate (Greiner Bio-One). In this design, 2 wells were used to produce 1 chamber with 1 inlet (culture well) and 1 outlet (i.e., 48 chambers available for cell culture). The outlet chamber was used to direct the effluent stream to exit through the top of the device so that the device bottom could be accessible for microscopy. As shown in Fig 1C, the culture device also consisted of: (1) 3 polydimethylsiloxane (PDMS) layers with each layer of 1 mm-thick, (2) polycarbonate (PC) membrane discs with 6 mm in diameter and 11 μm in thickness, (3) a 1.2 mm-thick glass layer, and (4) PDMS plugs. As illustrated in Fig 1B and 1C, the PDMS layers provided a fluidic channel of 2 mm thick and 6 mm wide between the inlet and outlet chambers and anchored the placement of the PC membrane discs in the culture chambers during the device assembly. The use of the membrane discs with 1μm pore size enabled: (1) the culture medium to perfuse through 3D tissue structures while trapping BMMC on the top surface of the discs and (2) optical access through the bottom of the culture chambers. Also, PDMS plugs located at the top of the culture chambers were used to facilitate the placement of cells into the culture wells at various time points during the experiments, as necessary. For the time-lapse microscopic observations of N-cadherin mediated adhesive interactions between BMMC and MMC, a PDMS-based microfluidic culture device consisting of hexagon-shaped culture chambers was used. Details associated with this device are described elsewhere.[11]

### BMMC co-cultured with 3D ossified tissue

The coculture preparation and cell maintenance has been previously described.[11] In brief, to build the 3D ossified tissue, 2x10^4^ human osteoblasts (hFOB 1.19, CRL-11372, purchased from American Type Culture Collection, ATCC, LGC Standards) were cultured in each culture well for 4 days prior the addition of BMMC. OSB growth medium (1:1 mixture of Ham's F12 Medium Dulbecco's Modified Eagle's Medium with 2.5 mM L-glutamine, supplemented with 10% fetal bovine serum (FBS), 0.3 mg/ml G418) was pumped into each chamber using a syringe pump (KDS230, KD Scientific).

Patient samples were collected with written consent in accordance with the Institutional Review Board (IRB)–approved protocol #00004259 at Hackensack University Medical Center (HackensackUMC), and in accordance with the Declaration of Helsinki. A set of the originally collected samples under protocol #00004259 were de-identified and used for this study after additional HackensackUMC IRB-approval (Protocol #00002811). BMMC and plasma were prepared from MM patient bone marrow aspirates by Ficoll-Paque gradient centrifugation. Patient disease information and sample usage for the various experiments conducted in this study are provided in Table 1. BMMC were frozen and stored in liquid nitrogen until use. Bone marrow culture medium (RPMI with L-glutamine, 10% patient plasma, 6.2x10^-4^ M of CaCl_2_, 1x10^-6^ M sodium succinate, 1x10^-6^ M hydrocortisone, 1 unit/ml heparin) was supplemented with 10% pooled plasma from 5 to 10 patients. For proliferation assays, BMMC samples were labeled with 0.5 μM (carboxyfluorescein diacetate, succinimidyl ester [CFSE], Life Technologies]). 4 to 8x10^4^ BMMC were introduced into the 3D ossified tissue culture chambers (Fig 1D). Flow was stopped to allow the settling of cells and reinstated after 4 h with the bone marrow growth medium. Perfusion cultures were kept in a humidified incubator at 37°C in 5% CO_2_. At the conclusion of the culture, cells were isolated from the chambers by trypsinization. The harvested tissue was further dissociated by grinding in microcentrifuge tubes. Single cell suspensions were prepared by filtering the grinded tissue through a 40 μm cell strainer.

**Table 1:** Patient Information.

Patient	Newly Diagnosed (N) or Relapsed (R)	Stage ^a^	Isotype	CD138^+^ MMC% in BMMC before Seeding	Experiment
1	N	III A	IgG κ	17.2%	OSB ALP activity
2	N	II A	IgG κ	0.27%	OSB viability effect and Giemsa staining
3	R	III B	IgG κ	1.40%	N-cadherin binding
4	N	N/A	IgG λ	9.3%	N-cadherin binding
5	R	III A	IgG κ	2.6%	Perfusion flow effect
6	R	I A	IgG κ	0.37%	Perfusion flow effect
7	N	III B	IgG κ	20.6%	Perfusion and plasma concentration effects
8	R	I A	IgG λ	0.17%	Perfusion and plasma concentration effects
9	R	III A	Non secretory MM	19.4%	Perfusion and plasma concentration effects

^a^ *Stage according to the Durie Salmon staging*.

### Alkaline phosphatase (ALP) activity staining

To evaluate the MM- mediated OSB suppression, OSB osteogenic phenotype change was assessed by measuring ALP activity in two types of cultures: OSB alone and OSB + BMMC coculture at three different coculture times: 0, 5 and 10 days (effectively Day 4, 9 and 14 of OSB culture since OSB were cultured for 4 days prior to the seeding of BMMC). The ALP activity was measured using a colorimetric method based on the conversion of p-nitrophenyl phosphate into p-nitrophenol. OSB alone culture and OSB + BMMC cocultured samples were trypsinized and harvested at coculture times of Day 0, 5 and 10, washed with PBS, and immersed into 0.1% Triton X-100 in PBS solution prior to freezing at -80°C. Following three freeze–thaw cycles, 20 μL of supernatant was taken from the samples and added into 100 μL of 1 mg/mL p-nitrophenyl phosphate substrate (Sigma-Aldrich) solution in diethanolamine substrate buffer (Thermo Fisher Scientific) incubated at 37°C for 1 h. The production of p-nitrophenol was measured by absorbance at 405 nm. The absorbance intensity of p-nitrophenol was reported as relative ALP activity in arbitrary units (a.u).

### Downregulation of OSB N-cadherin expression

Stable gene silencing of the N-cadherin gene (CDH-2) on the hFOB1.19 OSB cell line was achieved using Genecopoeia shRNA constructs, designed with a proprietary algorithm that scores potential sequences for efficient knockdown of the endogenous gene. The lentiviral delivery system was selected because it does not require a mitotic event for integration into the genome, therefore it can be used to infect both dividing and non-dividing cells, and stable silencing is passed to daughter cells. The manufacturer provided four shRNA sequences, individually cloned into the psiHIV-H1 vector along with a negative (CSHCTR001-HIV-H1) scramble control for this vector. Cells transduced with control vector will be termed mock-transduced herein. For delivery and stable incorporation of the shRNA constructs, a lentiviral system, pseudotyped with the VSV-G envelope protein, was used following manufacturer’s guidelines. Positive vectors and scramble control contained a GFP reported cassette for transduction efficiency reporting purposes. In brief, using Fugene 6 Transfection Reagent (Roche), 293T human embryonic kidney cells were co-transfected with the lentiviral packaging mix (Lenti-Pac HIV expression packaging kit, Genecopoeia) and Genecopoeia shRNA constructs specific for human CDH-2 or the control vector for mock knockdown of transcript in OSB cells. Over the next 72 h, the supernatants from the 293T cells containing the lentiviral particles for each construct were harvested multiple times by replacements of media over the cells, and the collection filtered. Transduction of hFOB1.19 was performed by spinoculation of lentiviral particles (1 h, 25°C, at 800 × g).

To determine the role of N-cadherin in mediating OSB-MMC cell-cell adhesive interactions, two types of coculture were performed: (1) mock- (negative control) transduced OSB + BMMC, and (2) N-cadherin knockdown OSB + BMMC. To track patient-derived cells, BMMC were labeled with either eFluro 670 [eBioscience] or anti-CD138-PE (a MMC marker). Using the PDMS-based microfluidic device, time-lapse bright field images and florescent images were taken to monitor OSB-BMMC and OSB-CD138^+^ MMC interactions. After 48 h of coculture, the tissue constructs with CD138-PE labeled MMC were trypsinized and tested using flow cytometry to evaluate MMC retention rate in coculture, as the ratio of CD138^+^ MMC (#PE^+^ cells)/OSB (#GFP^+^ cells).

### Determination of knockdown efficacy

N-cadherin knockdown efficacy was assessed by flow cytometry using CD325-PE (Biolegend, clone 8C11) staining and by western blotting. To this end, transduced cells were rinsed with 1XPBS followed by protein extraction with RIPA buffer (The Thermo Scientific) containing complete protease inhibitor mixture. Proteins were separated by SDS-PAGE and transferred to nitrocellulose membrane. The membrane was blocked with 5% nonfat dry milk in PBS with 0.1% Tween 20 and then incubated with the following primary antibodies: monoclonal anti-N-Cadherin (1:500) (BD Biosciences, clone 32/N-Cadherin), or anti-actin (1:1000) (Santa Cruz Biotechnology, clone I-19). Bound antibodies were detected by anti-IgG conjugated with horseradish peroxidase. Proteins were detected with ChemiDoc MP Imaging System (Bio-Rad). Densitometry was performed the ImageJ software. Each N-Cadherin signal was normalized with the actin signal intensity.

### Flow cytometry and cell proliferation measurements

Cell proliferation and viability was assessed at four different perfusion flow rates (0 μL/min (static), 0.25 μL/min, 0.8 μL/min, 2.5 μL/min), and at two different plasma compositions in the culture medium: 5% AB-human serum (Invitrogen) + 5% patient-derived plasma (5% Hu + 5% Pa), and 10% patient-derived plasma (10% Pa).

For each culture condition, flow cytometric analyses were used to quantify the percentage of MMC populations in BMMC, the expansions of MMC and BMMC, and the percentage of viable OSB and patient cells using 7-AAD staining. All staining were performed in staining buffer (phosphate buffer saline [PBS] containing 1% bovine serum albumin [BSA] and 0.02% sodium azide). The following fluorochrome-conjugated mAb were used for MMC phenotypic characterization: mouse antihuman CD138-APC (clone B-B4), CD38-PECy5 (clone LS198-4-3) or CD38-PECy7 (clone IB6) and CD56-PE-Vio770 (clone AF12-7H3). CD13 (an OSB surface marker, clone WM15) conjugated to PE was used to differentiate OSB from BMMC and MMC (which were CFSE^+^). All mAb were purchased from Beckman Counter, BD Pharmingen or Biolegend. A minimum of 10,000 events were acquired and analyzed using a Beckman Coulter FC500 flow cytometer and the CXP cytometer analysis software (Beckman Coulter).

CFSE^+^ cells in dot plots were gated as BMMC. CFSE^+^CD38^+^CD56^+^ cells, CFSE^+^ CD38^+^CD138^+^ and CFSE^+^CD56^+^CD138^+^ cells were gated and identified as MMC subpopulations. MMC proliferation was determined by analyzing the CFSE intensity, as previously described.[11] CFSE^-^CD13^+^ cells in dot plots were gated as OSB. CFSE^-^CD13^+^7-AAD^+^ were gated and identified as dead OSB. The percentage of MMC in the bone marrow aspirates (Table 1) ranged from 0.17% (patient 8) to 20.6% (patient 7). These percentages were diluted in the presence of OSB, which proliferated at a much faster rate than BMMC or MMC. Therefore, flow cytometric data from 3 separate cultures were pooled to increase counts in order to analyze MMC population expansion as a function of flow rate and plasma concentration.

### Giemsa staining

OSB alone and OSB + BMMC cocultures were analyzed following a 4-week period in the absence of OSB replenishment, or after OSB refilling (2 x 10^4^ OSB) on week 2 of coculture. Giemsa staining (Sigma-Aldrich) was performed to identify OSB, BMMC and MMC. At the conclusion of the cultures, cells were isolated from the chambers by trypsinization and the harvested tissues were further dissociated by grinding in microcentrifuge tubes. A droplet of cell suspension was adhered onto coverslips by centrifuging to perform staining and quantification analyses. To estimate OSB and MMC viability, total cell numbers of live OSB and MMC were counted using 6 different images, (with a total of 18 different fields), from each group.

### Statistical analyses

Cultures were run in triplicates. Flow cytometric data, ALP activity and MMC viability data from Giemsa Staining images were expressed as mean ± standard deviation of the percentage of MMC (from dot plots) obtained from two separate culture wells at each time point. For multiple comparisons (> 2 groups), one-way repeated measures ANOVA was conducted, followed by Tukey's multiple comparison test on individual pairs to determine significant differences among groups when ANOVA test was statistically significant. Student *t* test was used when comparing two groups. A p < 0.05 was considered significant. Prism (GraphPad software) was used to perform analyses.

## Results

### OSB activity and viability is suppressed in the presence of patient-derived BMMC, resulting in the loss of BMMC and MMC long-term viability

**Fig 2:**
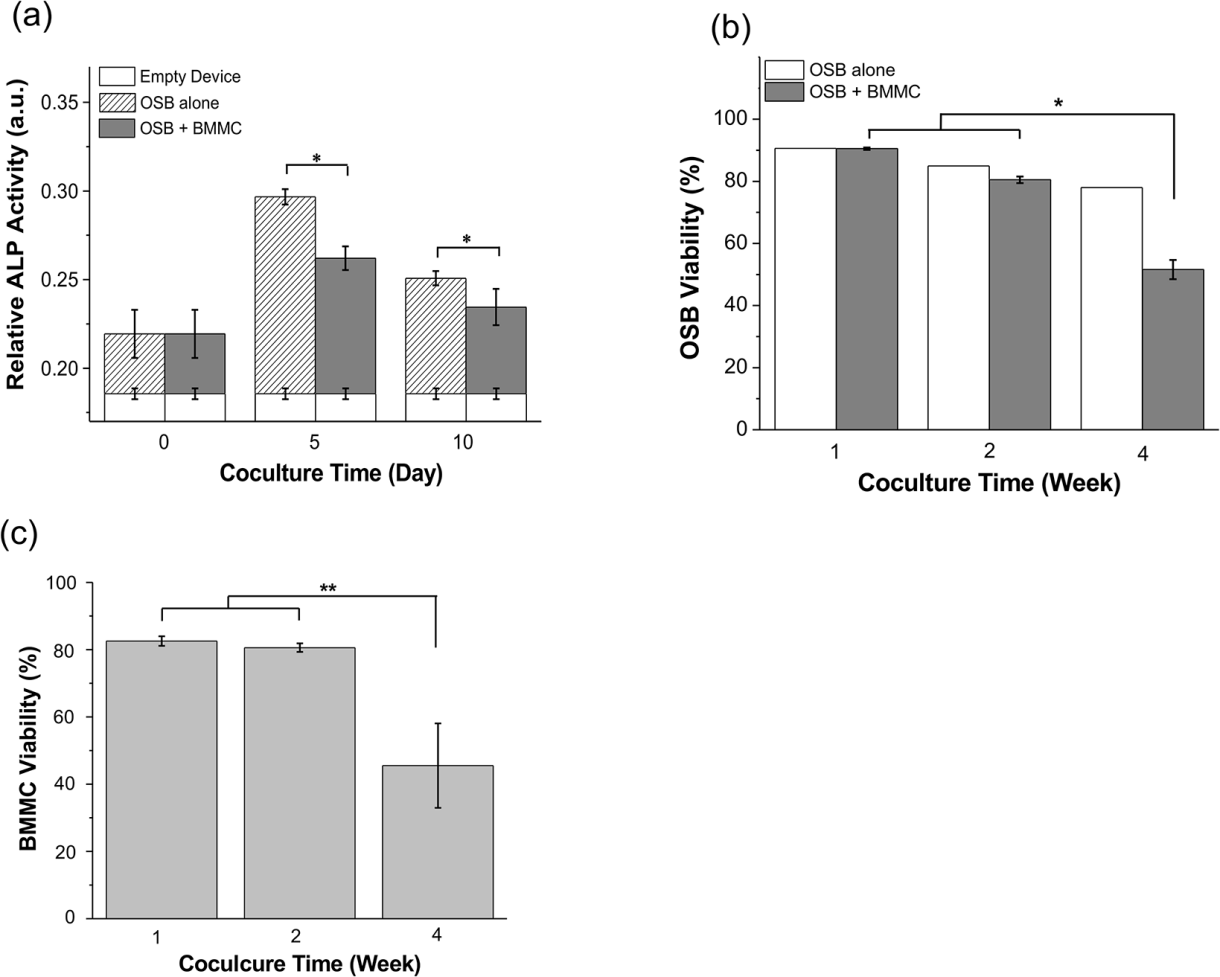
Suppressed OSB activity and viability, resulting in the loss of BMMC/MMC long-term viability. (a) Relative alkaline phosphatase (ALP) activity (a.u.) of OSB at Day 0, 5 and 10 of coculture. Taken into consideration the 4 day of OSB alone culture prior to BMMC seeding (in OSB culture medium), the actual culture time for OSB was 4, 9 and 14 as described in the methods section. (b) At each time point, OSB cell viability in coculture was assessed by co-staining cocultures with CD13 (an OSB marker) and 7-AAD to quantify dead OSB by flow cytometric analysis. ‘OSB alone’ bar is shown as reference. (c) Primary patient-derived BMMC were labeled with CFSE prior to coculture. BMMC cell viability was assessed by co-staining cultures with 7-AAD to quantify dead BMMC by flow cytometric analysis at indicated coculture time. * p < 0.05, ** p < 0.001.

To evaluate the effect of BMMC from MM patients on the osteogenic activity of OSB, we measured relative ALP production by OSB on Day 0, 5 and 10 of coculture (or since OSB were culture 4 days prior to adding BMMC, Day 4, 9 and 14 of OSB culture). As shown in Fig 2A, the production of ALP peaked on Day 5 in both OSB alone and OSB + BMMC cultures, but it was significantly lower in coculture; 0.297 ± 0.004 a.u. vs. 0.262 ± 0.007 a.u. (p = 0.026), respectively. This trend was also observed on Day 10, where relative ALP production for OSB + MMC culture was 0.235 ± 0.010 a.u. compared to 0.251 ± 0.004 a.u. in OSB alone cultures (p = 0.003). As shown in Fig 2B, OSB viability was evaluated after Week 1, 2 and 4 of coculture by co-staining tripsinized cultured tissues with CD13 (an OSB marker) and 7-AAD (which stains non-viable/dead cells). OSB viability in OSB + BMMC cocultures was significantly compromised after 4 weeks (51.59% ± 3.08%), compared to that at 1 week (90.54% ± 0.38%, p < 0.001) and 2 weeks (80.49% ± 1.03%, p < 0.001), respectively. Concomitantly, BMMC viability was also significantly decreased to 45.51% ± 12.56% after 4 weeks, compared to 82.57% ± 1.41% (p = 0.0076) and 80.60% ± 1.28% (p = 0.0092) viability, after 1 and 2 weeks in culture, respectively (Fig 2C).

### N-cadherin mediates adhesive interaction between OSB and MMC

**Fig 3:**
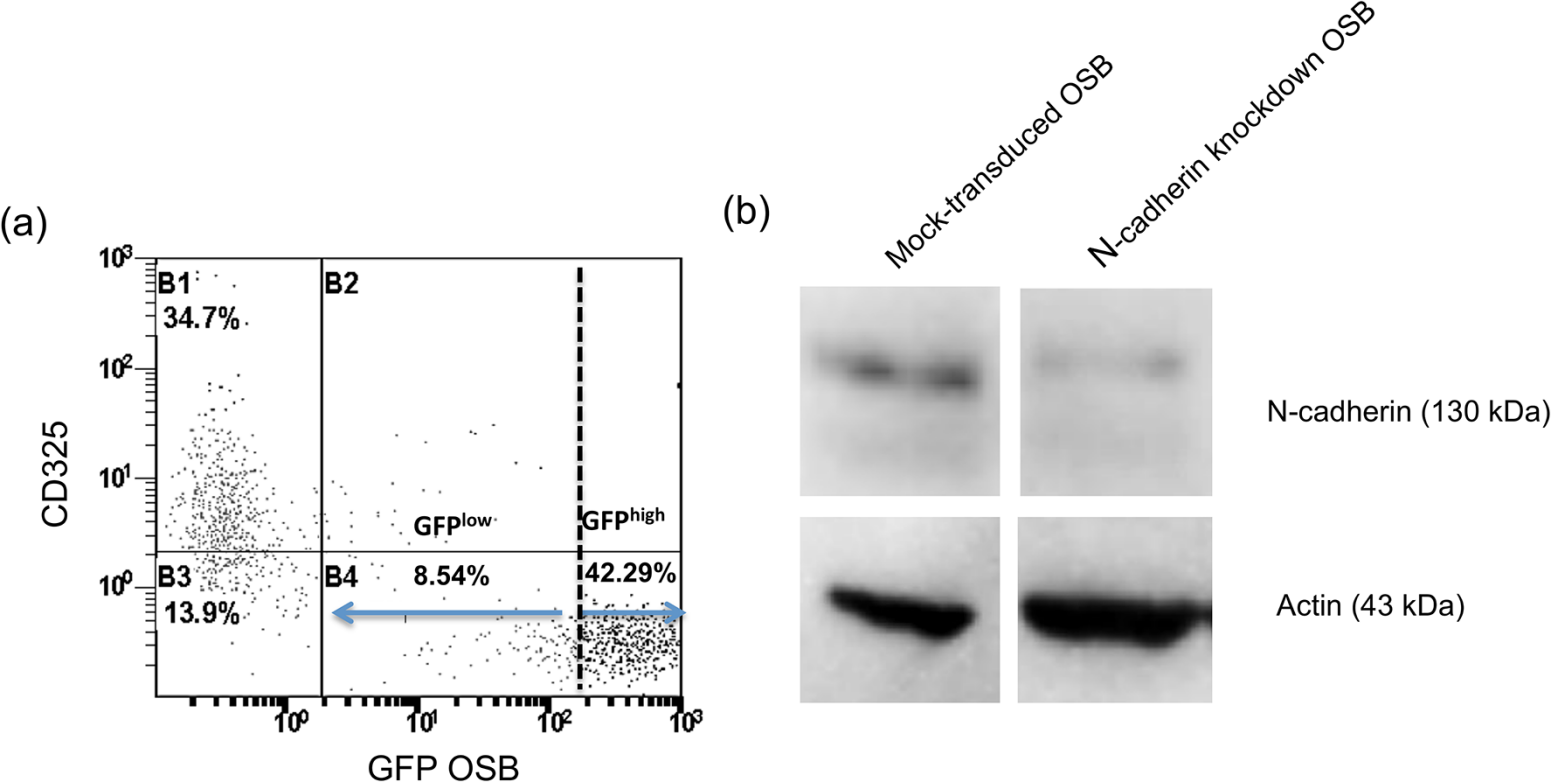
Downregulation of N-cadherin expression by hFOB1.19 OSB using shRNA lentiviral contsructs. (a) Dot plot representation of flow cytometric data showing reduced CD325 staining in shRNA-tranduced hFOB1.19 cells. GFPhigh cells (42.92%) were CD325-, 8.54% of the GFPlow cells still expressed N-cadherin, and 34.7% of transduced cells remained GFP-CD325+. (b) N-cadherin protein was also determined by Western blot analysis. Cell lysates were immunoblotted using monoclonal antibodies against N-cadherin and actin. Density of the bands of mock-transduced and N-cadherin knockdown hFOB1.19 of one representative experiment are shown.

OSB hFOB1.19 cells were transduced with shRNA lentivirus as described. Flow cytometric analysis (Fig 3A) showed that transduction efficiency was approximately 50%, as determined by the percentage of GFP^+^ OSB. The remaining GFP^–^ population was 34.7% CD325^+^ (Fig 3A). Western blot also confirmed the downregulation of N-cadherin (Fig 3B).

**Fig 4:**
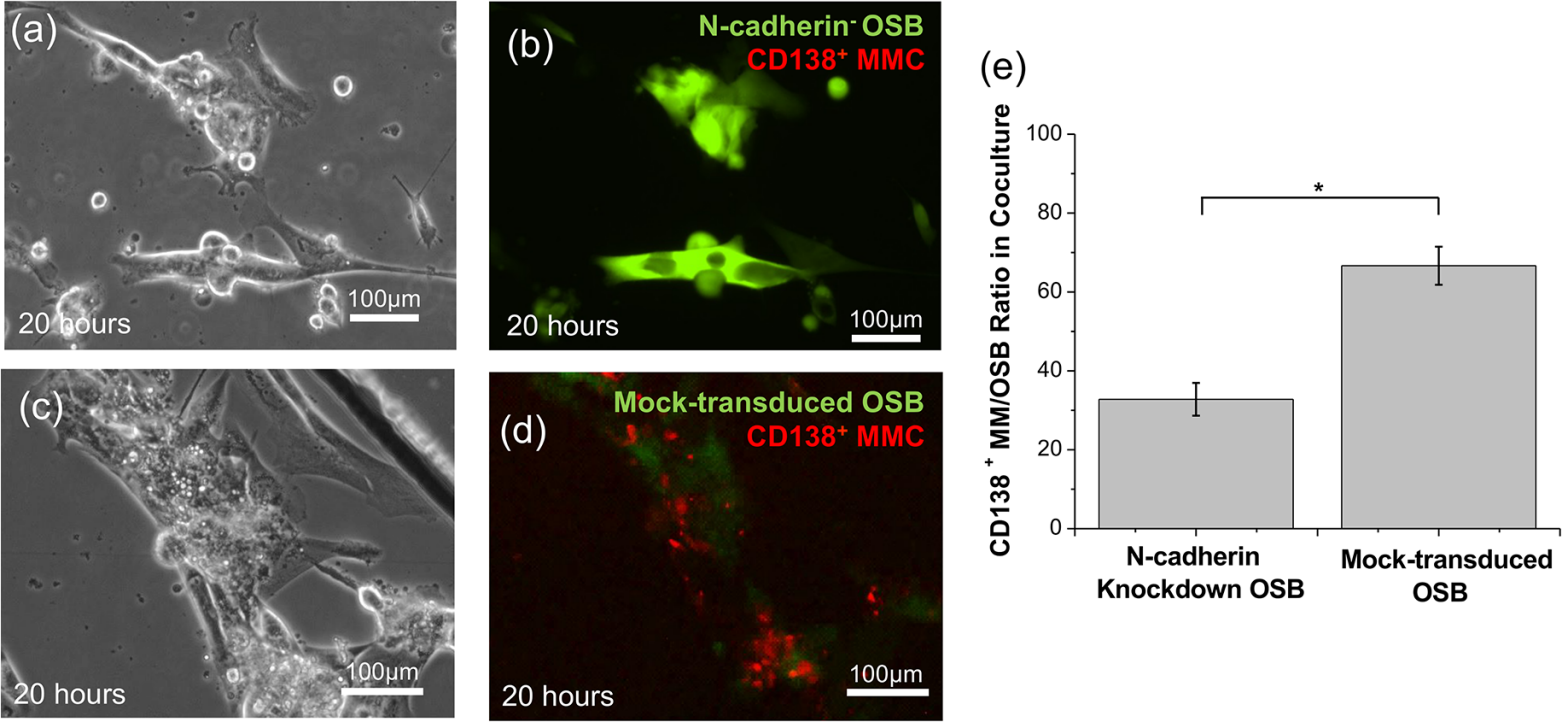
CD138^+^ MMC/OSB N-cadherin mediated interactions. Data presented is the response of patient sample #4 and it is representative of repeated experiments conducted using a total of 2 different patient samples (Table 1). (a-b) Bright field image and merged florescent image showing that CD138^+^ MMC (red, CD138 PE labeled) did not adhere to N-cadherin knockdown (green, GFP^high^) OSB. (c-d) Bright field image and merged florescent image of CD138^+^ MMC seeded onto mock-transduced OSB (green), showing that CD138^+^ MMC adhered to GFP^+^ mock-transduced cells. (e) Calculated retention ratio of CD138^+^ MMC seeded onto mock-transduced or N-cadherin knockdown OSB. After a 72 h coculture period, the retention ratio of CD138^+^ MMC was found to be significantly decreased when BMMC were seeded onto N-cadherin knockdown vs. mock-transduced OSB. * p < 0.05.

We then investigated the role of N-cadherin on the retention of BMMC and CD138^+^ MMC to the OSB scaffold. Real-time imaging was performed to evaluate the interaction of these cell populations as a function of N-cadherin expression by OSB, over a period of 72 h. The results of these experiments showed that CD138^+^ MMC could not adhered to GFP^+^ OSB in the N-cadherin knockdown OSB seeded chambers (Fig 4A and 4B) while CD138^+^ MMC attached to GFP mock-transduced OSB (Fig 4C and 4D). The retention of CD138^+^ MMC was tested by flow cytometry for CD138^+^ MMC/OSB ratio after BMMC was cocultured with both N-cadherin knockdown OSB and mock-transduced OSB. Notably, after 3 days of coculture, 70.21% ± 4.64% of CD138^+^ MMC were retained in the culture chambers seeded with mock-transduced OSB compared to a 32.78% ± 4.15% (p = 0.036) retention rate observed in the chambers layered with N-cadherin knockdown OSB (Fig 4E).

Using bright field imaging, we also observed that during the first 4 h of coculture while under static conditions, BMMC tended to migrate towards and adhered onto mock-transduced OSB. Such migrating tendency was not observed when BMMC were cocultured with N-cadherin knockdown OSB. On the other hand, BMMC attached uniformly throughout the mock-transduced OSB layer (S1 Fig).

### Perfusion flow rate effects on OSB and MMC viability

**Fig 5:**
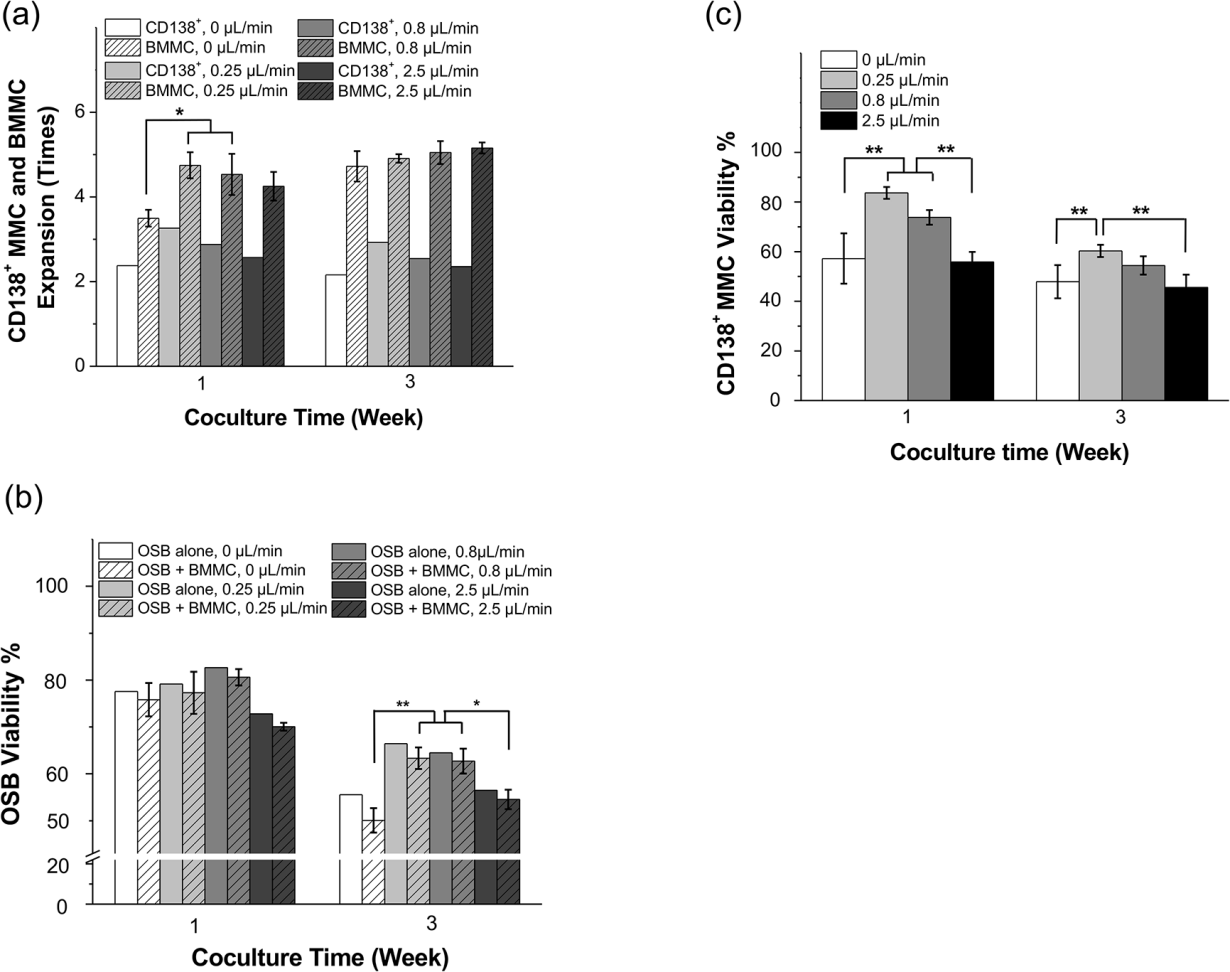
Effects of perfusion flow rate on OSB, BMMC and MMC viability. Data presented is the response of patient sample #8 and it is representative of repeated experiments conducted using a total of 5 different patient samples (Table 1). BMMC were labeled with CFSE prior to coculture in order to detect and quantify proliferation. Three different flow rates (0.25, 0.8 and 2.5 mL/min) were tested and compared to 0 mL/min (i.e., static condition). (a) Average expansion of BMMC and CD138^**+**^ MMC was calculated at indicated flow rates, using multi-color flow cytometric analysis. (b) OSB viability in coculture was determined by flow cytometric analysis of 7-AAD staining to quantify the percentage of dead OSB among CFSE^**–**^ cells. ‘OSB alone’ bar is shown as reference. (c) CD138^**+**^ MMC viability was also assessed by 7-AAD staining. * p < 0.05, ** p < 0.01.

To evaluate the role of media perfusion on the ex vivo survival of MMC, samples from 5 different patients were subjected to four different flow rates ranging from 0 μL/min (static condition) to 2.5 μL/min. Data plotted in Fig 5 is the response of one sample and it is representative of repeated experiments conducted on these 5 patient samples. Analyses of the expansion of the entire BMMC population and that of CD138^+^ (Fig 5A), CD38^+^CD56^+^, CD38^+^138^+^, CD56^+^CD138^+^ MMC subpopulations (S2 Fig) revealed that perfusion flow rates of 0.25 μL/min and 0.8μL/min resulted in the greatest BMMC expansion (4.75 ± 0.31 [p = 0.014] and 4.53 ± 0.49 [p = 0.033] times, respectively) compared to static culture conditions (3.50 ± 0.20 times).

Additional data from different patient samples (shown in S2 Fig) consistently showed that 0.25 μL/min and 0.8μL/min flow rates resulted in more BMMC expansion compared to static or 2.5 μL/min culture conditions. Likewise, MMC populations (Fig 5A and S2 Fig) followed the same expansion trend as BMMC. Among the MMC populations, CD38^+^CD138^+^ cells expanded as much as 3.3 times at 0.25 μL/min after 1 week.

OSB viability in OSB + BMMC coculutres (Fig 5B) was significantly higher at 0.25 μL/min (63.35 ± 2.30%, p = 0.002) and 0.8 μL/min (62.71 ± 2.65%, p = 0.002) compared to static conditions (50.08 ± 2.61%). However, viability at 0.25 μL/min flow rate (63.35 ± 2.30%, p = 0.012) and 0.8 μL/min (62.71 ± 2.65%, p = 0.017) was significantly greater compared to that at 2.5 μL/min (54.53 ± 2.07%) [S1 Table].

Concomitant with these results, viability of CD138^+^ MMC (Fig 5C) was also highest at 0.25 μL/min flow rate (83.66 ± 2.38% at 1 week, and 60.29 ± 2.44% at 3 weeks) compared to static conditions (57.23 ± 10.14% at 1 week [p = 0.003], and 47.91 ± 6.69% at 3 weeks [p = 0.004]), or compared to 2.5 μL/min (55.88 ± 3.98% at 1 week [p = 0.002], and 45.58 ± 5.14% at 3 weeks [p = 0.002]). p values for Fig 5A–5C are summarized in S1 Table.

### Effect of plasma concentration in the culture medium on MMC viability and proliferation

**Fig 6:**
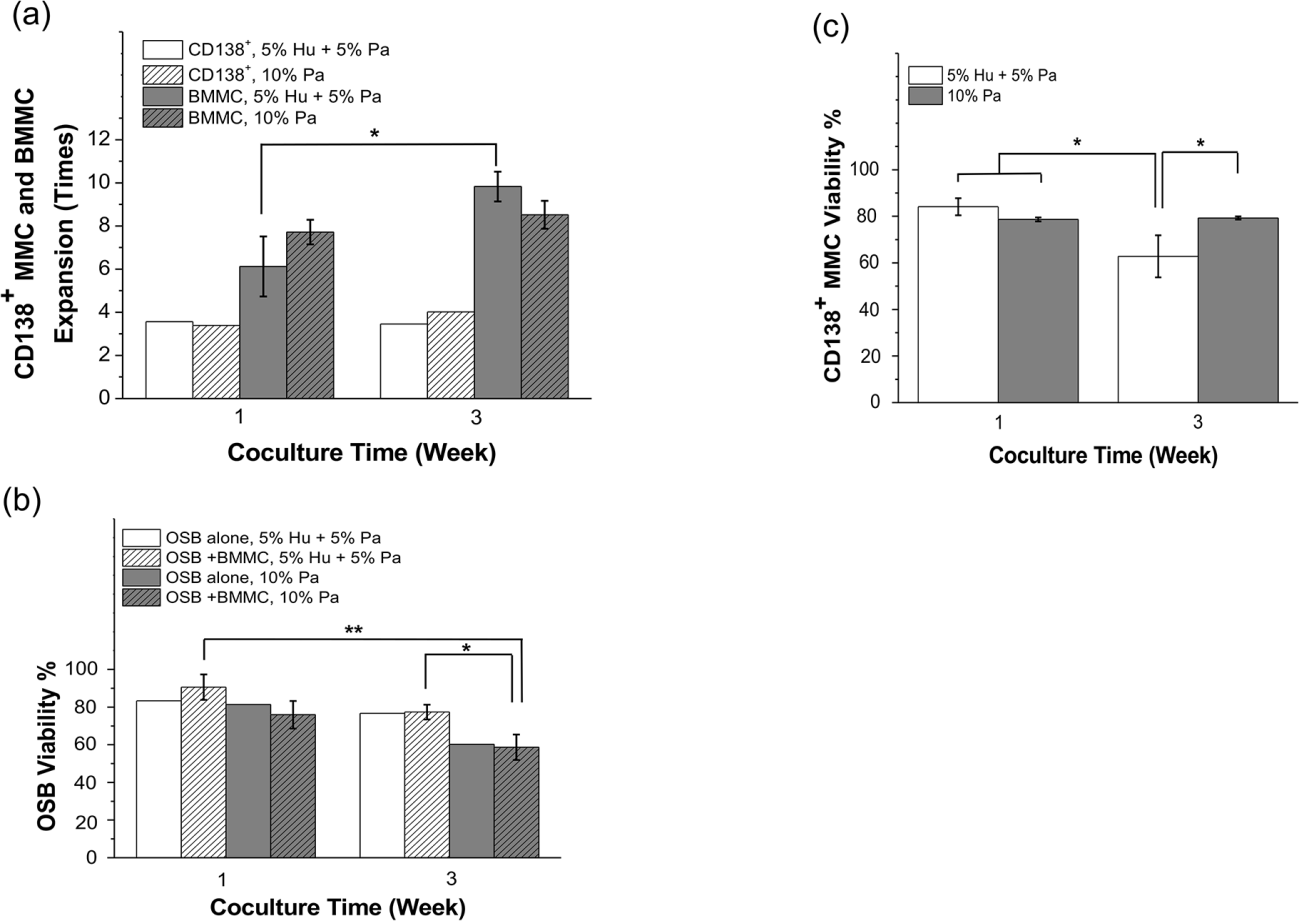
Effects of patient-derived plasma concentration in the culture medium on OSB and MMC viability. Data presented is the response of patient sample #10 and it is representative of repeated experiments conducted using a total of 3 different patient samples (Table 1). Patient-derived BMMC were labeled with CFSE prior to coculture in order to detect and quantify proliferation. Two different plasma concentration were tested: (1) 5% patient plasma (Pa) + 5% commercially available human AB plasma (Hu) and, (2) 10% Pa. (a) Average expansion of BMMC and CD138^**+**^ MMC were determined using multi-color flow cytometric analysis. (b) OSB viability in coculture was determined by flow cytometric analysis of 7-AAD staining to quantify the percentage of dead OSB among CFSE^**–**^ cells. ‘OSB alone’ bar is shown as reference. (c) CD138^**+**^ MMC viability was also assessed by 7-AAD staining. ‘OSB alone’ bar is shown as reference. * p < 0.05, ** p < 0.01.

Plasma concentration effects were tested at two different media compositions: (1) medium supplemented with 5% commercially available human AB serum (Hu) + 5% patient-derived plasma, and (2) medium supplemented with 10% patient-derived plasma. Data plotted in Fig 6 is the response of one patient sample and they are representative of repeated experiments conducted with a total of 3 different patient samples. CD138^+^ and CD38^+^CD56^+^, CD38^+^138^+^, and CD56^+^CD138^+^ MMC (Fig 6A and S3 Fig) exhibited an increasing expansion trend when the culture medium contained 10% Pa. In contrast, BMMC cells experienced significant expansion in culture medium supplemented with a mixture of 5% Hu + 5% Pa (6.11 ± 1.40 at Week 1 versus 9.83 ± 0.69 at Week 3, p = 0.005) (Fig 6A). Additional data from another patient sample also showed that: (1) 5% Hu + 5% Pa resulted in more BMMC expansion compared to 10% Pa and (2) 10% Pa resulted in more MMC expansion (S3 Fig).

OSB viability in OSB + BMMC cultures, on the other hand, was significantly compromised (58.68 ± 6.77%) after 3 weeks in the medium supplemented with 10% Pa compared 5% Hu + 5% Pa (90.61 ± 6.79% at 1 week, p = 0.004 vs. 77.37 ± 3.91% at 3 weeks, p = 0.04). On the contrary, CD138^+^ MMC viability was significantly decreased in cultures containing the 5% Hu + 5% Pa mixture. After 3 weeks, CD138^+^ viability was only 62.82 ± 9.06%, compared 79.25 ± 0.66% (p = 0.003) in cultures supplemented with 10% Pa. Furthermore, while MMC viability significantly decreased, between Weeks 1 and 3 in cocultures supplemented with 5% Pa (62.82 ± 9.06% vs. 84.12 ± 3.67%, p = 0.03), we observed no significant change over time with 10% Pa supplementation (78.66% ± 0.87% vs. 79.25%± 0.66%). Lastly, by week 3, CD138^+^ viability in 5% Pa containing medium was also significantly decreased compared to that obtained with 10% Pa at week 1 (62.82 ± 9.06% vs. 78.66 ± 0.87%, p = 0.04, Fig 6C). p values for Fig 6A–6C are summarized in S2 Table.

### Effect of OSB replenishment on the long-term viability and proliferation of MMC

**Fig 7:**
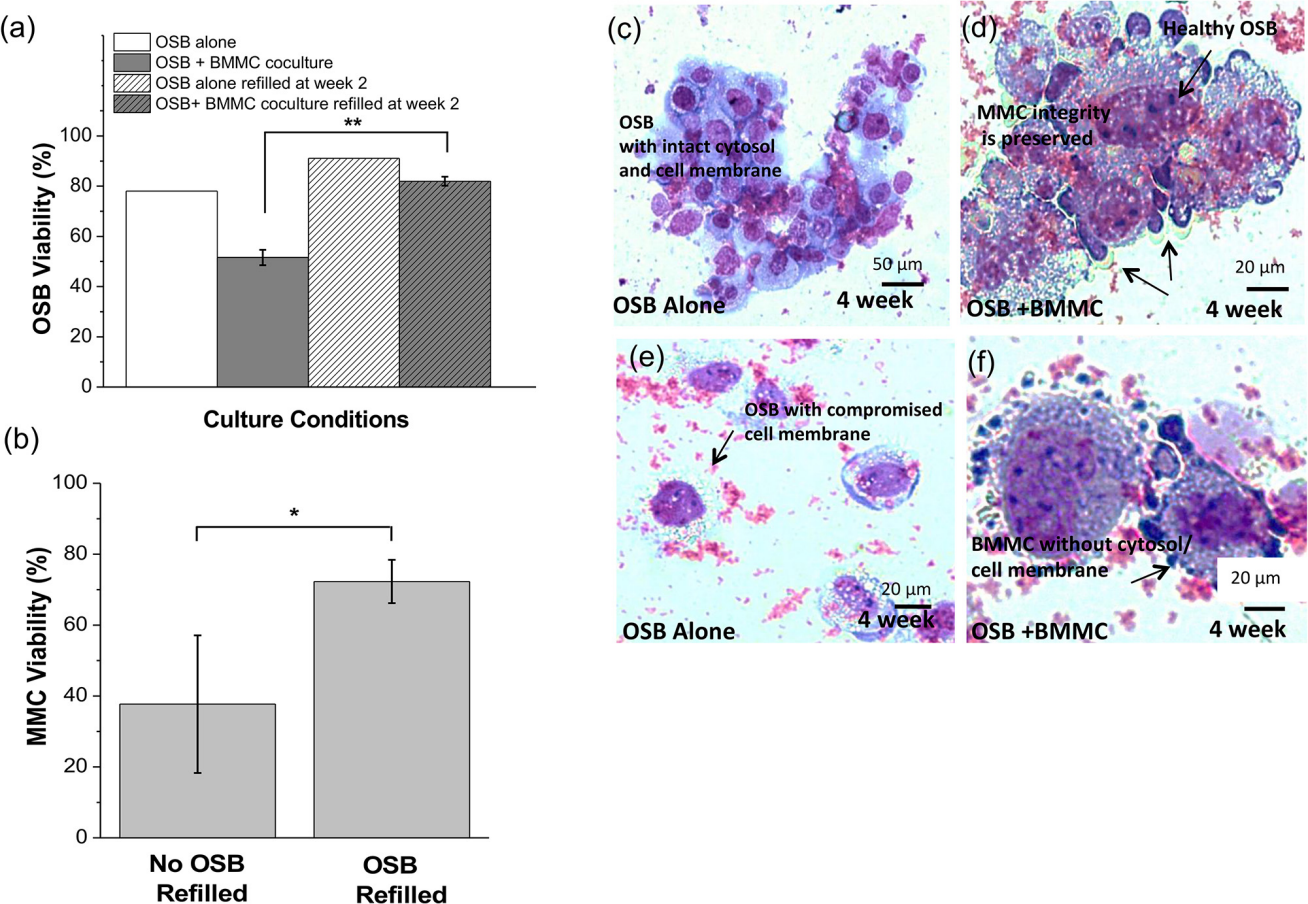
Effect of OSB replenishment on long-term ex vivo maintenance of MMC. (a) OSB cell viability in coculture was assessed by co-staining cultures with CD13 and 7-AAD to quantify dead OSB by flow cytometric analysis. ‘OSB alone’ bars are shown as reference. (b) MMC viability was evaluated by counting live and dead MMC cells (as determined by morphological analysis using Giemsa staining). Data presented is an average count of 18 separate fields. * p < 0.05, ** p < 0.01. (c-f) Giemsa staining of 4 weeks old cultures of OSB alone or OSB + BMMC coculture with (c-d) OSB refilled after 2 weeks, and (e-f) no replenishment.

To evaluate the effect of OSB viability on MMC growth, flow cytometric analysis of 7-AAD staining was performed on four different culture conditions after 4 weeks cocluture: (1) OSB alone, (2) OSB + BMMC, (3) OSB replenished on Week 2 of culture and (4) OSB + BMMC where OSB were refilled on Week 2 of culture. As shown in Fig 7A, 4-week OSB viability (i.e., 7-AAD^-^ cells) remained the highest on the chambers containing OSB only (78.03% without replenishment and 91.05% with replenishment). In OSB + BMMC chambers, refilling of OSB significantly improved the long termed viability of OSB (81.97% ± 1.78% in replenished chambers vs. 51.59% ± 3.08% in the absence of OSB supplementation, p < 0.001). More importantly, long term OSB viability was translated into a concomitant increase in MMC survival. As shown in Fig 7B, MMC viability was significantly higher in replenished vs. no replenished cultures (70.21% ± 4.64%, vs. 32.78% ± 4.15%, p = 0.008). A qualitative analysis of cell morphology and integrity (Giemsa staining, Fig 7C–7F) also confirmed that replenishment of OSB helped maintain the viability of both OSB and MMC. In the absence of OSB refilling (Fig 7E–7F), the remaining OSB appeared compromised (black arrow, Fig 7E) with many of the still adhered MMC and/or BMMC displaying a lack of cytosol integrity (black arrow, Fig 7F).

## Discussion

Using a microfluidic platform to emulate the physiology of the bone marrow microenvironment, we recently reported on the development of a new system to culture clinical bone marrow aspirates from MM patients. This approach has been designed with the intend to be use as a tool for personalized therapeutics, while providing a suitable template to study this disease using primary cells as opposed to cell lines or complicated and costly patient derived xenograft models. In this study we conducted additional experiments to: (1) evaluate the mechanism by which the long-term survival of OSB asserted the ex vivo survival and proliferation of primary human MMC; (2) systematically examine the effects of perfusion and plasma concentration on MMC-OSB interplay; and (3) test the feasibility of replenishing OSB during culture as a practical means of prolonging MMC’s viability and expansion beyond several weeks.

As part of the first aim, we investigated the role of N-cadherin in mediating BMMC/OSB and MMC/OSB interactions using, for the first time, genetically modified OSB with downregulated N-cadherin expression. Notably, as the GFP reported gene accompanied N-cadherin^-^ cells (Fig 3A), we could easily identify a propensity for both BMMC (S1 Fig) and CD138^+^ MMC (Fig 4A and 4B) to selectively attach to GFP^-^ OSB, suggesting that N-cadherin is, at least in part, responsible for BMMC/MMC and OSB adhesive interactions. As a result, approximately 70% of CD138^+^ MMC were retained in the chambers seeded with mock-transduced OSB compared to only 32.78% in chambers containing N-cadherin^-^ OSB.

OSB and MMC cell-cell adhesions have also been reported to be mediated via α4β1-VCAM-1 and/or homophilic N-CAM interactions.[21] However, an increasing number of studies have implicated the gaining of N-cadherin expression by solid tumors with an enhanced invasive potential and patient mortality.[24,26,27] Furthermore, Sadler, et al.,[23] recently showed, that inhibition of N-cadherin in MM cell lines cocultured with BM stromal cells or OSB played a critical role in regulating MM proliferation and viability. In particular, the authors noted that N-cadherin blocking resulted in massive death of the non-adherent MM cells while it led to a significant expansion of the MM stem cell population in the RPMI-8226 and U266 cell lines. In line with these reports, our results suggest that N-cadherin expression by OSB alone, is also contributing factor regulating the homing and/or retention of MMC to the tumor microenvironment.

This finding is important since there is emerging evidence to suggest that, in MM, direct adhesive interactions between malignant plasma cells and the bone marrow stroma are responsible for the development of drug resistance and relapse.[23,28] In other blood tumors such as leukemia, cancer stem cells in particular, have been shown to express N-cadherin and reside in a bone marrow niche that maintains them in a non-proliferative, drug-resistant state, thus, contributing to the maintenance of a cancer stem cell pool that when reactivated is responsible for tumor relapse.[23] This phenomenon termed "cell adhesion-mediated drug resistance" (CAM-DR) is also thought to be one of the major mechanisms by which MMC escape the cytotoxic effects of therapeutic agents.[29]

It has been also reported[22] that MMC N-cadherin-mediated interaction with OSB progenitor cells contributed to the ability of myeloma cells to inhibit osteoblastogenesis. For this reason, we investigated OSB ALP activity after coculture. ALP is a marker for early stage osteoblastic differentiation,[17] which declines before late stage differentiation as OSB start to produce mineralized extracellular matrix.[17,30] Consistent with the previous report, our results showed that ALP activity peaked on Day 5, started to decline after early cell differentiation,[17] and became suppressed in the presence of BMMC/MMC (Fig 2A). Both clinical histomorphometric analyses of bone samples from MM patients[31,32] and in vitro coculture studies using human MM cell lines [33] indicated that MMC inhibited OSB differentiation via downregulation of osteocalcin and collagen I. It has also been reported that in human osteoprogenitor cells, blocking of Runx2/Cbfa1 activity resulted in decreased OSB proliferation and activity,[32,34] suggesting multiple pathways could have also played a role in the observed declined in ALP activity in our coculture experiments.

Due to its confinement within bones, the marrow is subjected to a unique mechanical environment modulated by external factors such as physiological activity and disuse. These forces are determined among others by hydrostatic pressure, fluid flow induced shear stress, and viscosity.[35,36] Therefore, changes in the mechanical and compositional microenvironment of the bone marrow may affect the fate of resident cells, the homeostasis of bone, and consequently the activity of tumors residing within the constraints of the bone/bone marrow niche. The provision of perfusion-driven flow is a unique element present in our culture device. Hence, we were able to investigate the effects of flow rate on the viability and expansion of primary MMC.

The perfusion flow rate studies indicated that both MMC and OSB viability were greater at flow rates of 0.25 μL/min and 0.8 μL/min (Fig 5A–5C). These results suggest that these cells as well as their interactions respond to mechanical shear forces. It is known that shear stresses induced by fluid flow can stimulate human OSB cell proliferation and differentiation.[16,37] As OSB viability was found to be a prerequisite for the ex vivo maintenance of primary patient-derived MMC, we hypothesize that the appropriate perfusion to stimulate the proliferation and maintain functional OSB, along with cell-cell adhesion interactions between OSB and MMC, enabled the ex vivo viability and expansion of MMC (Fig 4A and 4C). We cannot however rule out the possibility that shear forces directly influenced MMC behavior and proliferation. In particular, work by Porat et al., showed that MM cell lines were differentially responsive to mechanical perturbations induced by shear flow and link this microenvironmental factor to the migratory potential of these cells,[35] indicating that MMC may be also equipped with mechanisms to sense and respond to shear forces.

The medium composition studies showed that patients’ plasma was clearly needed for the survival of MMC. In particular, while 10% Pa induced more MMC expansion (Fig 6A and S3 Fig), 5% Pa resulted in significantly greater BMMC expansion. Viability of CD138^+^ MMC was also higher when cultured in 10% Pa supplement medium (Fig 6C). However additional patient plasma in the culture medium induced more OSB death compared to 5% Pa, suggesting that cocultures with high plasma concentrations could also be counterproductive for the survival of MMC since it negatively affected OSB survival.

Tumor growth factors such as IL-6,[38,39] interleukin-1 beta (IL-1β), vascular endothelial growth factor (VEGF), insulin-like growth factor 1 (IGF-1), hepatocyte growth factor (HGF), tumor necrosis factor-α (TNF-α), Interleukin-10 (IL-10), Interleukin-15 (IL-15), Interleukin-21 (IL-21), and granulocyte macrophage colony-stimulating factor (GM-CSF), have all been found to promote MMC expansion.[39,40] It is also known that other secreted factors produced by MMC such as HGF, DKK1, IL-3, IL-7, TNF-α and the soluble-frizzled related protein-2 (SFRP2) can function as OSB inhibitors of differentiation and activity.[21] Together with published data demonstrating the suppressive role of the aforementioned inhibitory factors on stromal cells,[41,42] our results confirm the importance of optimizing the plasma concentration in the culture medium in order to support MMC without compromising OSB viability.

To support the long-term culture of MMC, healthy OSB were re-introduced in the system after a 2 week culture period to bypass the deleterious effects of MMC on OSB activity and viability (Fig 7A–7F). This strategy significantly extended the survival of viable MMC after 4 weeks coculture (Fig 7B). Furthermore MMC attached to healthy OSB presented with normal morphology (Fig 7D), whereas many dead BMMC/MMC lacking cytosol were observed in the chambers that were not replenished with healthy OSB on week 2 of coculture (Fig 7F).

**Fig 8:**
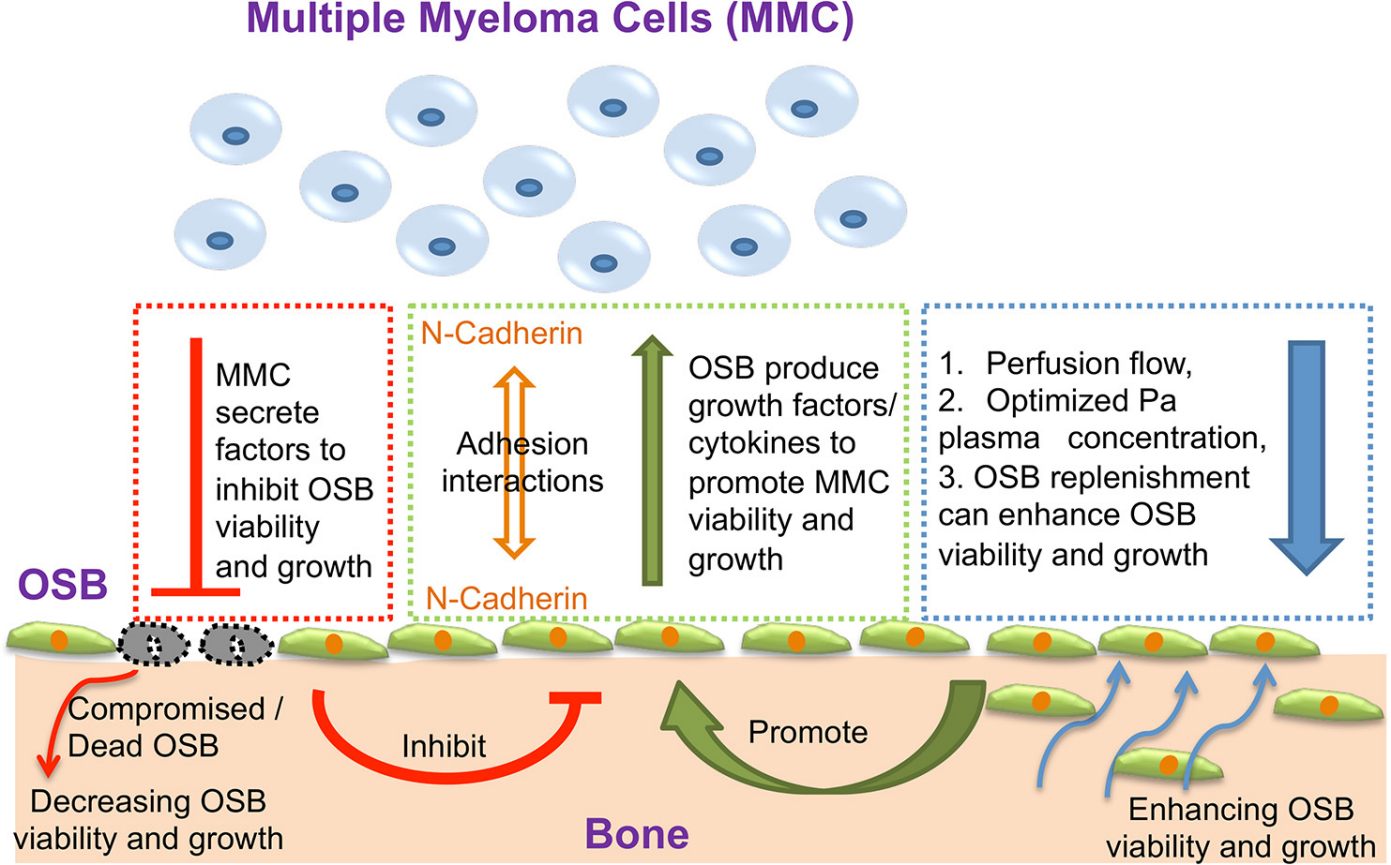
Optimized microenvironmental conditions and interactions required to support the ex vivo preservation of patient-derived MMC. It has been reported that MMC secrete soluble factors that can inhibit OSB proliferation [20] and induce OSB apoptosis. [17] MMC-OSB cell-cell interactions are mediated in part by N-cadherin homophilc interactions. [22, 29] Likewise, OSB support MMC growth by secreting a number of soluble factors. [18, 19] In this study, we showed that: (1) N-cadherin expression by OSB can mediate adhesion between MMC and OSB, (2) optimized perfusion flow and patient-derived plasma concentration are critical for the viability of both OSB and MMC, and (3) replenishing OSB during culture can be used a practical means of prolonging MMC viability and expansion.

From the culture device perspective, our results (summarized in Fig 8) demonstrate the possibility of: (1) replicating the multi-cellular MM tumor niche with perfusion as an important microenvironmental factor and (2) characterizing cell responses using conventional flow cytometry and imaging/staining techniques.

Future clinical implications of this study are expected to rely on the robustness of the developed platform. We demonstrated the preservation of a wide range of biospecimens from both newly diagnosed and relapsed patients, with an equally broad content of MMC. This is particularly important because devastatingly, most MM patients experience one or more relapses with the development of drug resistance. At relapse, drug evaluation is expected to be most critical because once patients become drug resistant they’ll likely required a more precise assessment of potential therapies, while balancing efficacy and toxicity. Importantly, our model incorporates many of the protective BM elements present within the tumor niche enabling MMC drug resistance, much like it occurs in vivo.

With this high-throughput device and optimization of the MM microenvironment niche device, we anticipate that this ex vivo tumor engineering approach may provide a new avenue for: (1) testing of personalized therapeutics for MM patients, (2) evaluation of new drugs without the need for costly animal models, and (3) studying the biology of MM and in particular the mechanisms responsible for drug resistance and relapse. Beyond multiple myeloma, our approach could be extended to study other osteolytic and osteoblastic diseases, which characterize the progression of metastatic tumors that reside within the constraints of the bone marrow microenvironment.

## Conclusions

In this study we found, for the first time, that the long-term survival of OSB is a critical factor maintaining the ex vivo viability and proliferative capacity of MMC. In this regard, we found that N-cadherin expressed by OSB contributes to the homing and/or retention of MMC to the OSB scaffolding. We also found that the long-term survival of both OSB and primary MMC could be enhanced by: (1) optimizing perfusion flow rate and patient-derived plasma composition in the culture medium, and (2) replenishing OSB during culture as a practical means of prolonging MMC’s viability beyond several weeks. By studying the microenvironmental factors that affect MMC localization, survival and growth, our results suggest that the optimized microenvironment to better recapitulate the bone marrow malignancy milieu, can allow maximum ex vivo MMC viability and expansion.

## Supporting Information

S1 Fig Data from patient 3 for BMMC/OSB N-cadherin mediated interactions. (a, b, c, e) Bright field images 4 h and 60 h after BMMC seeding into chambers containing either mock-transduced or N-cadherin knockdown OSB. (a) BMMC remained unattached to OSB in N-cadherin knockdown OSB seeded chambers (red arrows). (b) BMMC were found adhered to mock-transduced OSB (white arrows). (c-d) Bright field image and merged florescent image of BMMC (red, eFluro 670 labeled) showed that BMMC did not adhered to N-cadherin knowckdown (green, GFP^high^) OSB. (e-f) Bright field image and merged florescent image of BMMC (red) seeded onto mock-transduced OSB (green), showing that BMMC adhered to GFP^+^ mock-transduced OSB.
(TIFF)

S2 Fig Effects of perfusion flow rate on MM populations and BMMC expansion for patients 7 and 11. Patient-derived BMMC were labeled with CFSE prior to coculture in order to detect and quantify proliferation. Three different flow rates (0.25, 0.8 and 2.5 ul/min) were tested and compared to 0 ul/min (i.e., static condition). Cell expansion were calculated at indicated flow rates, using multi-color flow cytometric analysis. (a) Average expansion of patient #7 CD138^+^ MMC and BMMC. (b) Average expansion of patient #11 BMMC. Average expansion of (c) patient #8 and (d) patient 11’s MMC populations. * p < 0.05.
(TIFF)

S3 Fig Effects of patient-derived plasma concentration in the culture medium on MM populations and BMMC expansion for patients 9 and 10. Patient-derived BMMC were labeled with CFSE prior to coculture in order to detect and quantify proliferation. Two different plasma concentration were tested: 5% Pa + 5% Hu and 10% Pa. Cell expansion were calculated at indicated plasma concentration, using multi-color flow cytometric analysis. (a) Average expansion of patient 9 ‘s CD138^+^ MMC and BMMC. Average expansion of (b) patient #9 and (c) patient #10 MMC populations. * p < 0.05.
(TIFF)

S1 Table Statistical Analysis for Effects of Perfusion Flow Rate on OSB and MMC Viability from Fig 5. (TIFF)

S2 Table Table Statistical Analysis for Patient-derived Plasma Concentration in The Culture Medium on OSB and MMC Viability from Fig 6. (TIFF)
