# Supplementary figures and images for: Ex Vivo Maintenance of Primary Human Multiple Myeloma Cells through the Optimization of the Osteoblastic Niche
Source: PLoS One. 2015 May 14;10(5):e0125995. doi: 10.1371/journal.pone.0125995 (PMC4431864; doi:10.1371/journal.pone.0125995)

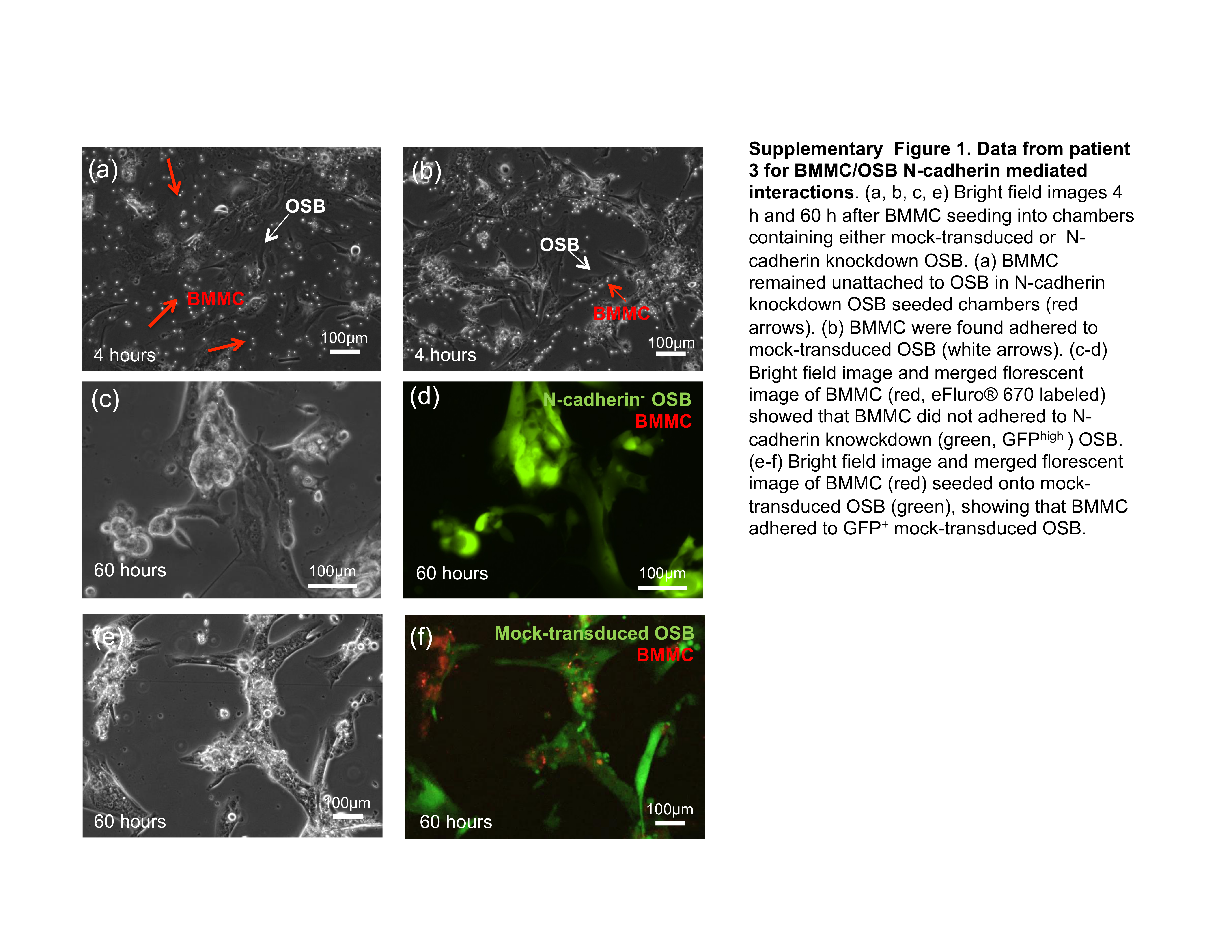

Supplement: S1 Fig — (a, b, c, e) Bright field images 4 h and 60 h after BMMC seeding into chambers containing either mock-transduced or N-cadherin knockdown OSB. (a) BMMC remained unattached to OSB in N-cadherin knockdown OSB seeded chambers (red arrows). (b) BMMC were found adhered to mock-transduced OSB (white arrows). (c-d) Bright field image and merged florescent image of BMMC (red, eFluro 670 labeled) showed that BMMC did not adhered to N-cadherin knowckdown (green, GFPhigh) OSB. (e-f) Bright field image and merged florescent image of BMMC (red) seeded onto mock-transduced OSB (green), showing that BMMC adhered to GFP+ mock-transduced OSB. (TIFF) [file pone.0125995.s001.tiff]

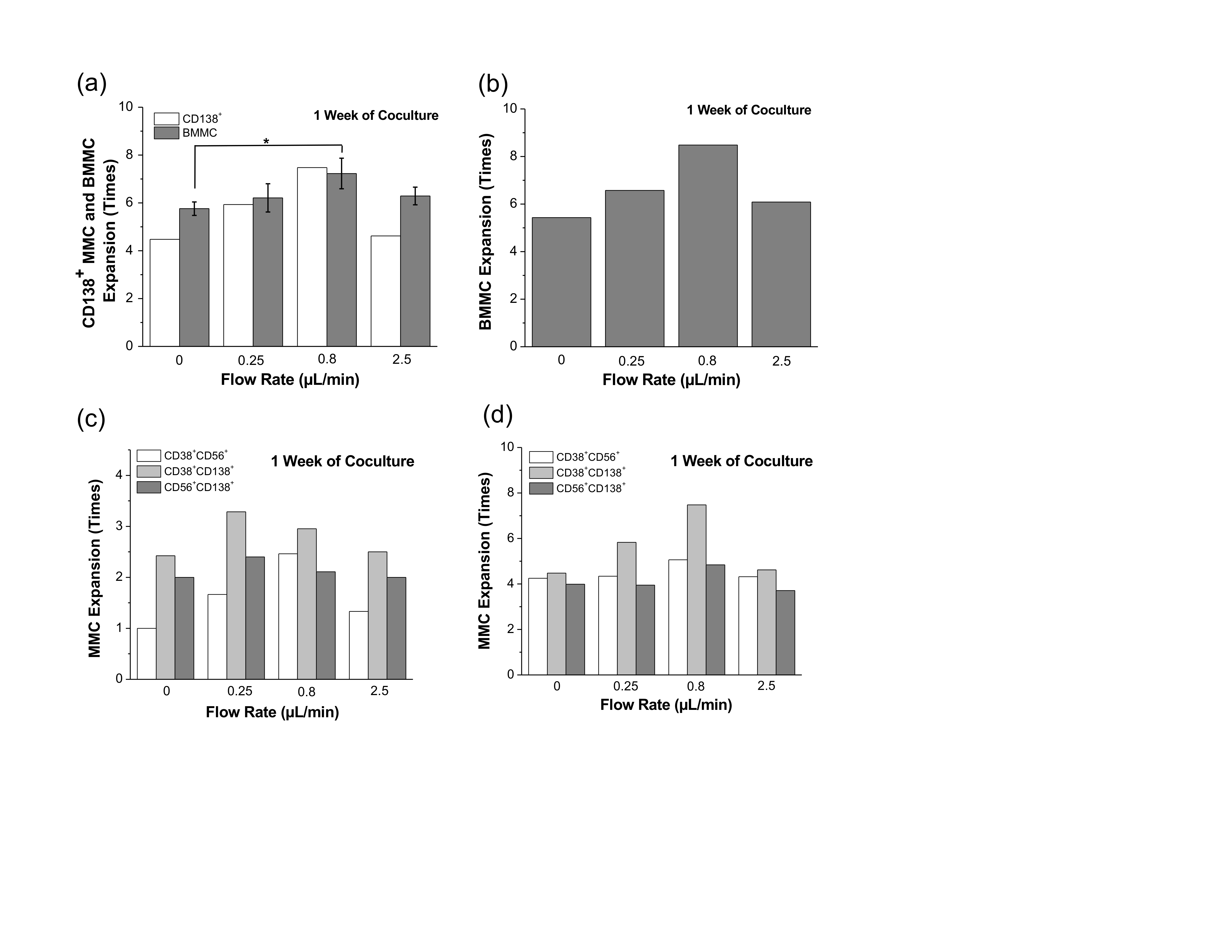

Supplement: S2 Fig — Patient-derived BMMC were labeled with CFSE prior to coculture in order to detect and quantify proliferation. Three different flow rates (0.25, 0.8 and 2.5 ul/min) were tested and compared to 0 ul/min (i.e., static condition). Cell expansion were calculated at indicated flow rates, using multi-color flow cytometric analysis. (a) Average expansion of patient #7 CD138+ MMC and BMMC. (b) Average expansion of patient #11 BMMC. Average expansion of (c) patient #8 and (d) patient 11’s MMC populations. * p < 0.05. (TIFF) [file pone.0125995.s002.tiff]

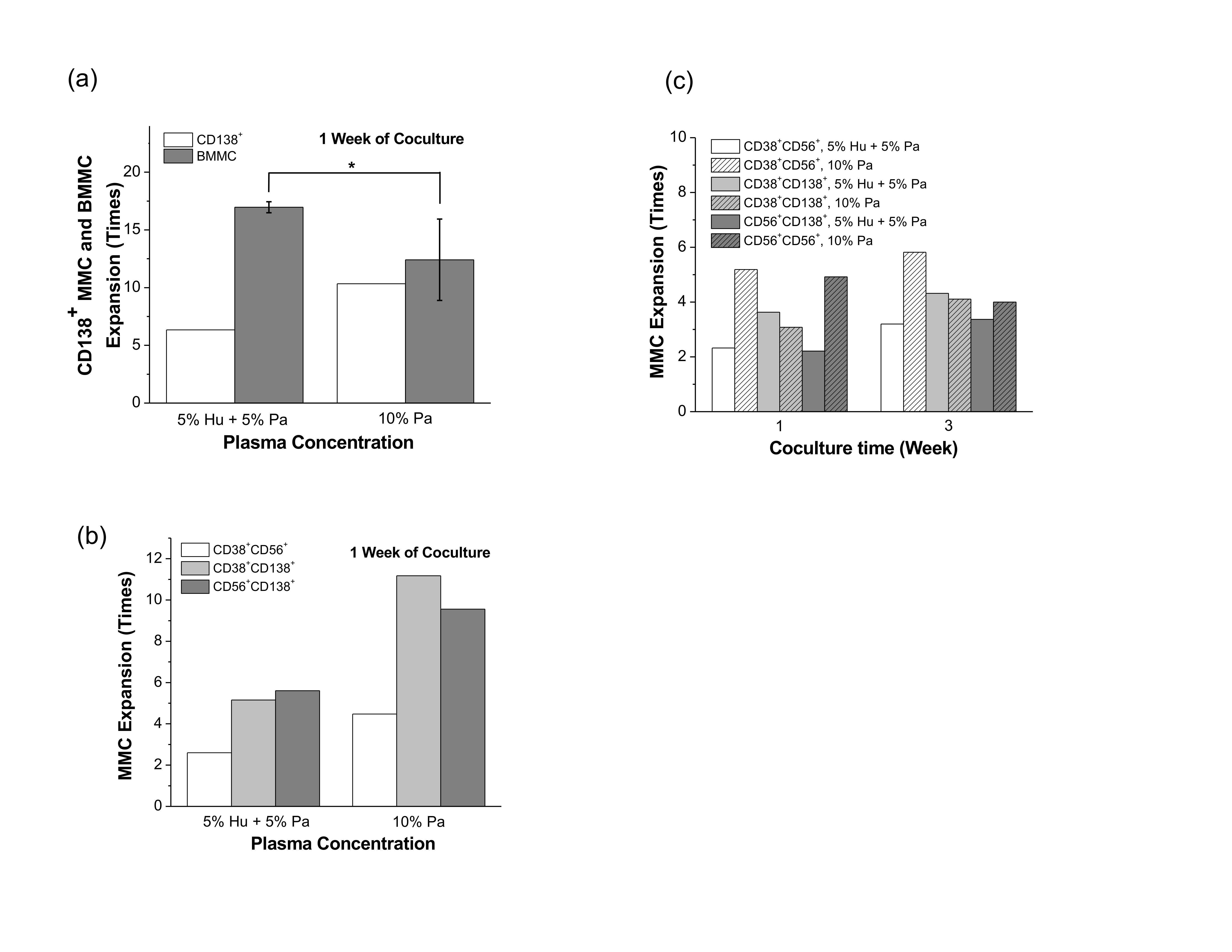

Supplement: S3 Fig — Patient-derived BMMC were labeled with CFSE prior to coculture in order to detect and quantify proliferation. Two different plasma concentration were tested: 5% Pa + 5% Hu and 10% Pa. Cell expansion were calculated at indicated plasma concentration, using multi-color flow cytometric analysis. (a) Average expansion of patient 9 ‘s CD138+ MMC and BMMC. Average expansion of (b) patient #9 and (c) patient #10 MMC populations. * p < 0.05. (TIFF) [file pone.0125995.s003.tiff]

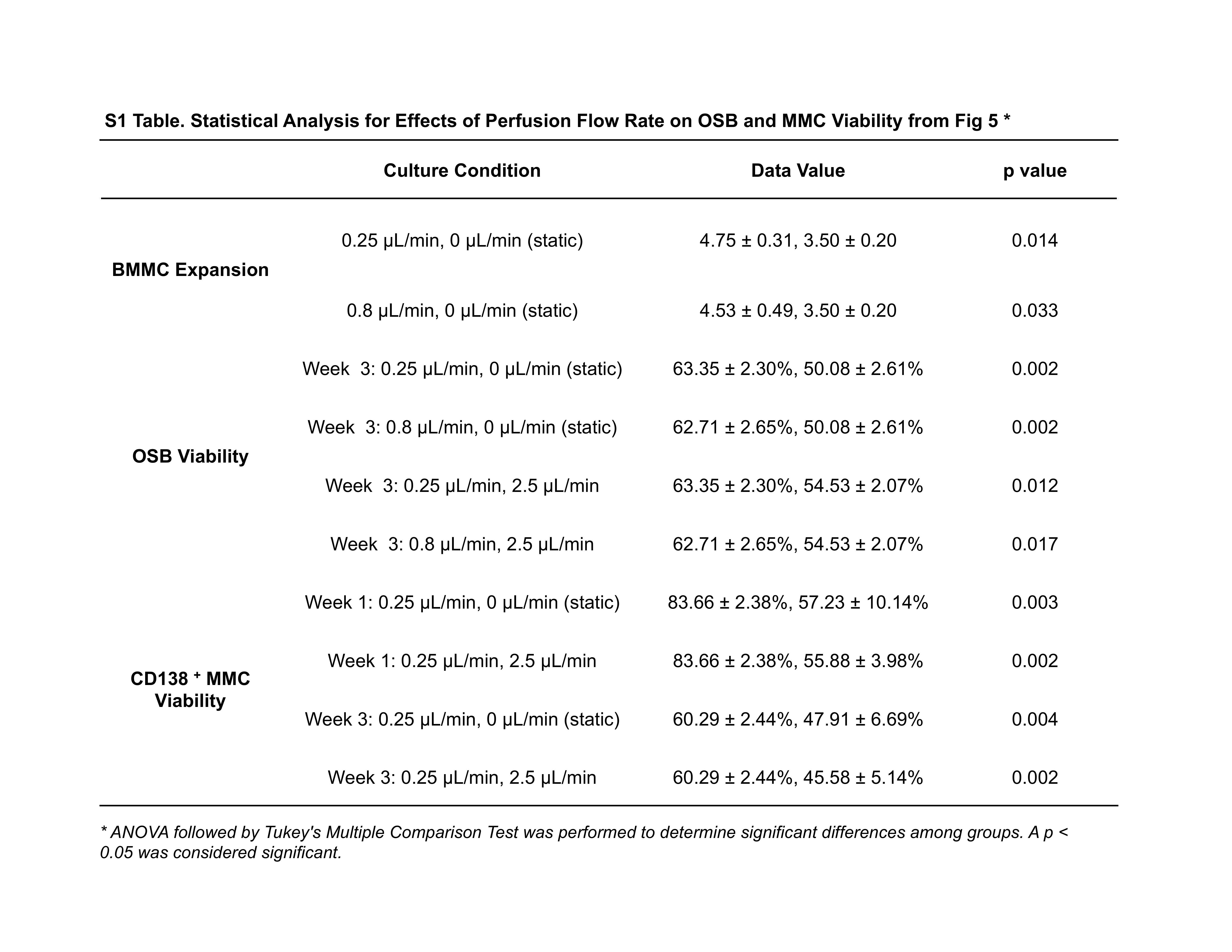

Supplement: S1 Table — (TIFF) [file pone.0125995.s004.tiff]

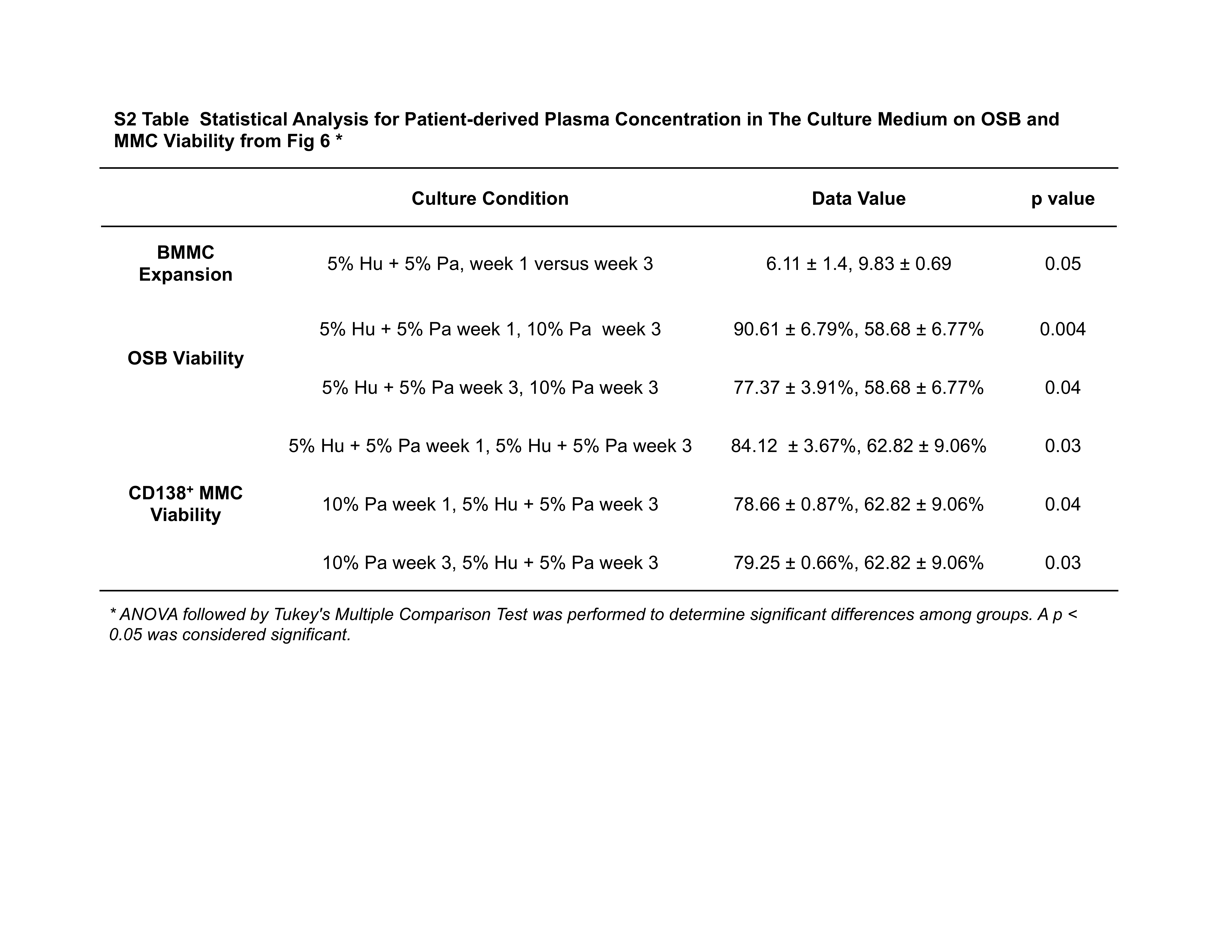

Supplement: S2 Table — (TIFF) [file pone.0125995.s005.tiff]
